# Neuropsychiatric Disorders and Burn Trauma: Insights From a Tertiary Care Hospital

**DOI:** 10.7759/cureus.72421

**Published:** 2024-10-26

**Authors:** Imran Ahmad, Nimisha Singh

**Affiliations:** 1 Plastic Surgery, Jawaharlal Nehru Medical College Hospital (JNMCH) Aligarh Muslim University, Aligarh, IND; 2 General Surgery, Himalayan Institute of Medical Sciences, Dehradun, IND; 3 Plastic and Reconstructive Surgery, Jawaharlal Nehru Medical College Hospital (JNMCH) Aligarh Muslim University, Aligarh, IND

**Keywords:** burn injuries, cerebrovascular disease, epilepsy, mental disabilities, neuropsychiatric disorders, parkinson’s

## Abstract

Highlights: Burn injuries pose a significant risk for individuals with neurological disorders, particularly during altered states of consciousness. This study investigated the relationship between various neurological disorders and burn injuries in a tertiary care hospital setting.

Materials and methods: This prospective observational study was conducted over 24 months at a university-affiliated tertiary care hospital. Medical records of patients with burn injuries attributed to neuropsychiatric causes were analyzed. Data collected included demographic details, neurological cause, burn severity, anatomical distribution, and management.

Results: The study revealed a higher prevalence of burn injuries among females (47, 54.76%) compared to males (37, 45.23%). While there was no statistically significant difference in the duration of neuropsychiatric disorders between males (8.5 ± 2.8 years) and females (10.43 ± 3.94 years) (p = 0.321), epilepsy was the most common neurological disorder (56, 66.52%), followed by mental disability (15, 17.85%), dementia (6, 7.15%), cerebrovascular disorders (5, 5.95%), and Parkinson's disease (3, 3.57%). Females experienced a higher percentage of scald burns (26.19%) compared to males (29.76%) (p = 0.012) and a higher proportion of third-degree burns (24.4% vs. 11.9% in males) (p = 0.010). Anatomically, burns were most prevalent on the head and neck (26, 30.95% females vs. 14, 31.1% males) (p = 0.003), upper extremities (16, 19.04% females vs. 17, 20.23% males), and anterior trunk (18, 21.40% females vs. 11, 24.4% males). Common interventions included debridement and dressings (17, 31.1% females vs. 14, 20.23% males), tangential excision with split-thickness skin grafting (18, 21.40% females vs. 16, 19.04% males), and conservative dressings (9, 10.71% females vs. 5, 5.90% males). Females had a longer mean duration of hospital stay (13.65 days ± 4.68) compared to males (10.54 days ± 3.27).

Conclusion: Neuropsychiatric disorders, particularly epilepsy, mental disability, and dementia, significantly contribute to burn injuries among affected individuals. This underscores the importance of targeted interventions in seizure management, home safety, and comprehensive medical care. Multidisciplinary collaboration and culturally informed strategies are essential for addressing this public health challenge effectively and improving patient outcomes.

## Introduction

Cerebral disorders, which cause psychiatric symptoms, are called neuropsychiatric disorders. The primary characteristics of neuropsychiatric symptoms include the simultaneous presence of various psychiatric symptoms with cognitive impairment being a central feature. These symptoms can manifest early as cerebral symptoms and sometimes resemble endogenous psychiatric disorders. Neuropsychiatric symptoms include anxiety, neurotic complaints, apathy, mood disorders, hallucinations, delusions, behavioral and personality changes, delirium, and cognitive impairment (dementia) [1]. On the other hand, mental disability is a condition that significantly impairs an individual's cognitive, emotional, or behavioral functioning, leading to difficulties in managing daily activities, maintaining relationships, and achieving personal goals. It includes a wide range of disorders, such as intellectual disabilities, mental health disorders (like depression, anxiety, and schizophrenia), and neurodevelopmental disorders (such as autism spectrum disorder and attention deficit hyperactivity disorder) [2].

Burn injuries pose a significant global public health challenge due to their frequent occurrence and potential for devastating physical, emotional, and financial impacts on individuals, families, and communities. These injuries often occur during altered states of consciousness associated with organic neurological diseases [3]. It is estimated that around 85% of individuals with neurological conditions live in low- and middle-income countries and may not seek conventional treatment due to cost or cultural beliefs [4]. The unpredictable onset of seizure activity and the resulting inability of patients to protect themselves from heat sources contribute to an increased risk of burns among epileptic patients, leading to more severe injuries [5].

Head injuries resulting from birth complications, falls, road traffic accidents, or interpersonal violence are the primary causes of seizure disorders worldwide. Additionally, endemic infections such as neurocysticercosis, falciparum malaria, schistosomiasis, HIV, toxoplasmosis, tuberculosis, meningitis, and encephalitis, along with inadequate management of childhood febrile illnesses, significantly contribute to the prevalence of other neurological disorders [6]. Prolonged exposure to heat during the onset of unconsciousness preceding an epileptic seizure can lead to severe and life-threatening burn injuries [7,8]. An epileptic seizure typically lasts for two to five minutes, during which time significant deep burns can occur. This is due to the fact that exposure to a heat source exceeding 71°C for one to two seconds can lead to a full-thickness skin injury [9]. Positive family history, extreme old age, and metabolic disorders can also contribute to the development of neuropsychiatric disorders such as meningitis, mental disability, and dementia.

The care of patients with major burn injuries is resource-intensive and often requires treatment at specialized centers, with profound impacts not only on the patient's life but also on caregivers and families. This study was conducted to explore the connection between neuropsychiatric disorders and burn injuries, underscoring the importance of creating and supporting targeted interventions in developing nations to address these critical health challenges.

## Materials and methods

This study aims to investigate the association between neuropsychiatric disorders and the characteristics of burn injuries, including their types, severity, anatomical distribution, surgical interventions, and clinical outcomes.

The prospective observational study was conducted at the Department of Plastic and Reconstructive Surgery, Jawaharlal Nehru Medical College, Aligarh Muslim University, India, from March 2022 to February 2024. The Ethics and Research Advisory Committee of the Faculty of Medicine of Jawaharlal Nehru Medical College, Aligarh Muslim University, approved the study (approval number: EC/NEW/INST/2020/904). During this period, 1,170 burn patients presented, including 84 with neuropsychiatric disorders related to their injuries. Cerebral disorders, which cause psychiatric symptoms, are called neuropsychiatric disorders, while mental disability is a condition that significantly impairs an individual's cognitive, emotional, or behavioral functioning, leading to difficulties in managing daily activities, maintaining relationships, and achieving personal goals. It includes a wide range of disorders, such as intellectual disabilities, mental health disorders (like depression, anxiety, and schizophrenia), and neurodevelopmental disorders. Medical records of patients with neuropsychiatric disorders sustaining burns were examined, and CT scans, electroencephalography, and disease-specific investigations were performed to confirm diagnoses. Data collected encompassed demographic details, neurological causes, burn severity, anatomical distribution, type of burn, and management strategies. This study aimed to elucidate the relationship between neuropsychiatric disorders and burn injuries, providing insights into effective management strategies for this complex patient population.

The inclusion criteria ensured that the study captured a broad spectrum of adult patients affected by burn injuries associated with neurological and psychiatric disorders. Exclusion criteria comprised patients who demonstrated non-compliance with treatment regimens, pregnant females, children, and individuals with high-tension electric burns (>1000V).

The data entry process was performed using Microsoft Excel 2010 (Microsoft Corporation, Redmond, WA, USA). Sample characteristics were described using descriptive statistics, including mean, standard deviation (SD), frequency, and percentage. Statistical analyses were conducted using SPSS Statistics version 22 (IBM Corp. Released 2013. IBM SPSS Statistics for Windows, Version 22.0. Armonk, NY: IBM Corp.). The Chi-square test was used to assess significant associations between categorical variables (percentage and frequency), while the t-test was employed to compare means of quantitative variables. A significance level of p < 0.05 was chosen to determine statistical significance in this study.

## Results

The majority of patients with burn injuries due to neuropsychiatric disorders belonged to lower socioeconomic status, according to the revised Kuppuswamy socioeconomic status classification (2021). Out of the 1,170 burn patients, 84 (7.2%) had co-existing neuropsychiatric disorders, highlighting the significant overlap between burn injuries and neurological conditions. Among the 84 patients with neuropsychiatric disorders, the average age of males was 38.43 years (SD = 10.32), while females had an average age of 34.27 years (SD = 11.54). The male-to-female ratio was 1:1.25, with 37 males (44%) and 47 females (56%) among the 84 patients. This distribution allows for a comprehensive analysis of neuropsychiatric disorders and associated burn injuries across both sexes. Table 1 shows the sex-wise comparison of various parameters related to neuropsychiatric disorders.

**Table 1:** Sex-wise comparison of various parameters related to neuropsychiatric disorders N: number of participants, %: percentage, SD: standard deviation, a: t-test, b: Chi-square test

Neurological disorder		Male N (%)	Female N (%)	p-value
Mean duration (mean ± SD) in years		8.5 ± 2.8	10.43 ± 3.94	0.321^a^
Type	Epilepsy	27 (32.00%)	29 (34.52%)	0.214^b^
	Mental disability	6 (7.14%)	9 (10.71%)	
	Dementia	3 (3.57%)	3 (3.57%)	
	Stroke	1 (1.19%)	4 (4.76%)	
	Parkinson diseases	2 (2.38%)	1 (1.19%)	
Cases (diagnosed)	New	18 (21.42%)	21 (25%)	0.127^b^
	Old	20 (23.8%)	25 (29.76%)	
Medication adherence	Regular	12 (26.00%)	14 (31.1%)	0.532^b^
	Irregular	8 (17.7%)	11 (24.4%)	

In this study, the mean duration of neurological disorders did not show a significant difference between males (8.5 ± 2.8 years) and females (10.43 ± 3.94 years), with a p-value of 0.321, indicating comparable durations of neurological conditions between the sexes. Similarly, the distribution of specific neuropsychiatric disorders did not significantly vary between males and females (p = 0.214), suggesting a similar prevalence of these conditions across genders. The study also examined the diagnosis status, finding no significant difference in the distribution of new versus old cases between males and females (p = 0.127), indicating similar rates of new diagnoses among both groups. Furthermore, medication adherence, whether regular or irregular, did not differ significantly between males and females (p = 0.532), highlighting comparable adherence levels to prescribed treatments across genders in the study cohort. Table 2 shows the sex-wise comparison of various parameters related to burns.

**Table 2:** Sex-wise comparison of various parameters related to burns N: number of participants, %: percentage, STSG: split-thickness skin graft, BKA: below-knee amputation, *: statistically significant (p < 0.05)

Burns		Male N (%)	Female N (%)	p-value
Type	Scald burns	20 (23.80%)	18 (21.42%)	0.012*
	Flame burns	8 (9.52%)	4 (4.76%)	
	Thermal	2 (2.38%)	5 (5.95%)	
	Domestic	3 (3.57%)	12 (17.85%)	
	Chemical	5 (5.95%)	7 (8.33%)	
Etiology	Accidental	25 (29.76%)	29 (34.52%)	0.015*
	Homicidal	11 (13.09%)	13 (15.47%)	
	Suicidal	2 (2.38%)	4 (4.76%)	
Source of injury	Stove burn	9 (10.71%)	22 (26.19%)	0.03*
	Hot liquid	7 (8.33%)	10 (11.9%)	
	Natural gas	4 (4.76%)	4 (4.76%)	
	Hot metal	8 (9.52%)	3 (3.57%)	
	Gasoline	3 (3.57%)	2 (2.38%)	
	Electricity	2 (2.38%)	3 (3.57%)	
	Corrosive ingestion	4 (4.76%)	2 (2.38%)	
Degree of burns	Second degree	28 (33.33%)	35 (41.86%)	0.010*
	Third degree	10 (11.9%)	11 (13.09%)	
Anatomical distribution	Head and neck	14 (16.66%)	26 (30.95%)	0.003*
	Upper extremities	17 (20.23%)	16 (19.04%)	
	Lower extremities	7 (8.30%)	4 (4.76%)	
	Anterior trunk	11 (13.09%)	18 (21.40%)	
	Posterior trunk	4 (4.76%)	5 (5.90%)	
	Genital	7 (8.30%)	9 (10.71%)	
Intervention	Tangential excision and STSG	16 (19.04%)	18 (21.40%)	0.192
	Debridement and dressings	14 (31.1%)	17 (20.23%)	
	Amputation: BKA	3 (3.57%)	2 (2.38%)	
	Conservative dressings	5 (5.90%)	9 (10.71%)	

The dataset provides valuable insights into the demographic distribution and contextual vulnerabilities associated with burn injuries, highlighting important sex-wise disparities and variations across different etiologies and sources of burns. It was found that females exhibited a higher overall prevalence of burn injuries compared to males (p = 0.012). While scald burns affected both sexes similarly, males comprised 20 (23.80%) of the 84 patients and females 18 (21.42%) (p = 0.012). However, domestic burns showed a significant female predominance, with 15 females (17.85%) compared to three males (3.57%) (p < 0.001). Homicidal burns, though less common, exhibited a slightly higher prevalence among females, with 13 cases (15.47%) compared to 11 males (13.09%) (p = 0.25). Accidental burns were more prevalent among females, with 29 cases (34.52%) compared to 25 males (29.76%) (p = 0.015), suggesting potential differences in exposure or risk perception. Females showed significant differences in burn distribution, particularly in the head and neck regions, upper extremities, and anterior trunk, with higher prevalence rates compared to males (p = 0.003). Females presented a higher proportion of third-degree burns, with 20 females (24.4%) compared to 10 males (11.9%). Both genders underwent tangential excision and split-thickness skin grafting at comparable rates. Rates of amputation (below-knee amputation) showed no significant gender difference. Females had a longer mean duration of hospital stay (13.65 ± 4.68 days) compared to males (10.54 ± 3.27 days), suggesting potentially more complex cases or slower recovery among females and higher mortality.

## Discussion

Over the past several years, significant advancements have been made in the management of burn injuries. However, in developing nations, particularly in rural areas, many individuals still rely on inadequate treatment options, leading to increased comorbidities and disabilities. This contributes to higher rates of sepsis, organ damage or failure, and even mortality. Addressing these challenges requires improving healthcare infrastructure, promoting preventive measures, enhancing healthcare quality, and addressing social determinants of health to mitigate the impact of these factors on morbidity rates and improve overall population health. Patients with neuropsychiatric diseases often receive insufficient attention in these regions, contributing to a higher prevalence of burn injuries among affected individuals.

In a study by Ibrahim et al., it was found that women constituted the majority of burn cases among individuals with neurological disorders [10], a trend that aligns with our study, where females accounted for 47 (54.76%) of cases compared to 37 (45.23%) among males. In contrast, Mohammadi et al. found that the majority of patients with epilepsy who developed burn injuries were males [11]. In rural areas, due to the lack of resources, women are predominantly engaged in household chores, compared to men, who are more involved in other work [10]. The mean age for females was 34.27 ± 11.54 years, while for men, it was 38.43 ± 10.32 years, consistent with the study by Jang et al. [12]. The mean duration of the neurological disorder was 8.5 ± 2.8 years for males and 10.43 ± 3.94 years for females. There were a total of 21 females and 18 males with newly diagnosed cases of neuropsychiatric disorders. In this study, seizure disorder (56, 66.52%) remained the most common neuropsychiatric disease, followed by mental disability (15, 17.85%), dementia (6, 7.15%), cerebrovascular disorders (5, 5.95%), and Parkinson's disease (3, 3.57%).

In this study, 38 (55.95%) of the patients suffered from scald burns, followed by 12 (22.46%) with flame burns, seven (8.33%) with thermal burns, and the remaining injuries from domestic current, consistent with the study by Jang et al. [12] and Wang et al. [13]. Females showed a significantly higher prevalence of stove burns (26.19% vs. 10.71% in males, p = 0.03), potentially linked to gender-specific behaviors or exposures. Other burn sources (e.g., hot liquids, hot metals, and natural gas) demonstrated less pronounced sex-based disparities, indicating a more uniform distribution across sexes. However, burns due to corrosive ingestion were more common in males (4, 4.76%) than in females (2, 2.38%). In a study by Zia Ziabari et al., 40% of patients sustained second-degree burns, while 22.5% suffered third-degree burns [14]. In this study, 63 patients (75%) had second-degree burns, and 21 patients (25%) had third-degree burns (p = 0.010). This study suggests a higher prevalence of accidental burns among females (29, 34.52%) compared to males (25, 29.76%) (p = 0.015), potentially reflecting varied exposures or risk perceptions. Homicidal burn rates were slightly higher in females (15.47% vs. 13.09%), and females also showed a trend toward more suicidal burns (4.76% vs. 2.38%), warranting further investigation. Most patients in this study experienced a loss of consciousness, resulting in prolonged contact with the offending agent and deeper burns. The most common sites involved were the upper extremities, including the hand (33, 31.2%), followed by the head and neck (40, 22.9%), anterior trunk (29, 19.6%), and lower extremities (11, 19.6%), while the least involved were the genitalia (16, 6.5%) and posterior trunk (9, 5.3%). Yang et al. reported that the most common burn site was the upper limbs, accounting for 61.96% of all cases, followed by the lower limbs (52.72%) [13]. Patients, especially women engaged in household chores, often fall onto the offending agent after losing consciousness, which could explain the increased involvement of the head, neck, and upper extremities, followed by the anterior trunk.

Among the interventions, 30 (25.76%) of the patients underwent serial debridement and dressings, while 34 (20.4%) underwent tangential excision and split-thickness skin grafting. Fourteen (8.3%) of the patients were managed conservatively with various silver dressings. Five (2.9%) had to undergo below-knee amputation of the affected limb, as it could not be salvaged and was a source of severe sepsis. In a study by Jang et al., the amputation rate ranged from 4.3% to 19.0% [12]. Wang et al. [13] stated that the most common type of surgery was autologous skin grafting, accounting for 71.74%, followed by amputations of the hand, accounting for 19.02%. Two patients (2.3%) out of 84 succumbed due to other comorbidities, consistent with the results of a study by Zia Ziabari et al., which reported a mortality rate of 7.5% [14]. Females had a longer mean duration of hospital stay (13.65 ± 4.68 days) compared to males (10.54 ± 3.27 days), suggesting a higher degree of burn, as increased depth of burns requires more surgical intervention and care, similar to the results of a study by Li et al. [15].

Burn injuries are not limited to patients with seizure disorders; other neuropsychiatric conditions such as Parkinson's disease, cerebrovascular disorders, schizophrenia, and severe mental disability also pose significant risks. These conditions can impair motor coordination, judgment, and awareness of surroundings, making individuals more prone to accidents, including burn injuries. For instance, patients with dementia may wander into hazardous environments or fail to recognize dangers such as open flames or hot surfaces, while those with severe psychiatric illnesses may be involved in self-harm or violent incidents leading to burns. Moreover, neurodegenerative disorders like Parkinson’s can result in muscle stiffness and reduced mobility, which increases the likelihood of burn injuries due to falls or the inability to react swiftly to dangerous situations. Primary prevention strategies must be prioritized, including environmental modifications like securing hot surfaces, using safety equipment, and installing fire alarms. Seizure control measures, such as proper medication adherence and regular monitoring, play a key role in reducing the risk of burn injuries in patients with epilepsy. Additionally, caregiver education on seizure first aid, as well as home safety adaptations, are essential for creating safer living environments for these individuals. Preventing burn injuries associated with neuropsychiatric disorders requires a comprehensive approach focused on seizure management and home safety. Consistent and effective management of seizures, including medication adherence and regular medical supervision, is paramount to reducing seizure-related risks [16]. Burn scars, functional limitations, and dependence on others for care can exacerbate feelings of shame, guilt, and low self-worth, particularly in communities where disabilities are stigmatized. Culturally informed interventions, including community awareness campaigns and targeted counseling, are paramount to addressing the social stigma associated with both burn and neuropsychiatric disorders. Regular medical check-ups are necessary to monitor neuropsychiatric disorders and optimize treatment plans [17]. Additionally, providing psychological support to patients and families can help them cope with the challenges of living with neuropsychiatric disorders. By implementing these preventive measures comprehensively, the incidence and severity of burn injuries related to neurological diseases can be significantly reduced, improving overall safety and quality of life for affected individuals.

This study had the following limitations: its single-center setting and small sample size limit the generalizability of its findings to broader populations. Additionally, reliance on patient interviews and medical history may introduce inaccuracies in reporting neurological history and the circumstances of the burns. The study primarily focused on a few neuropsychiatric disorders, potentially overlooking other conditions that may also increase the risk of burn injuries.

## Conclusions

The correlation between neuropsychiatric disorders and burn injuries emphasizes the critical need for targeted interventions, particularly to reduce seizure activity. Among the 1,170 burn patients examined, 84 individuals (7.2%) had co-existing neuropsychiatric disorders, underscoring a significant overlap between these conditions. A large proportion of these patients were from lower socioeconomic backgrounds, further highlighting the vulnerability of this population. Seizure disorder was the most prevalent neurological condition among these patients, accounting for 66.52% of cases, followed by mental disability (17.85%) and dementia (7.15%). Females exhibited a higher proportion of burn injuries compared to males, particularly with stove burns and domestic accidents. Anatomically, females suffered more severe burns, including a higher proportion of third-degree burns, and experienced longer hospital stays. The higher severity of burns and extended recovery periods in females also led to increased mortality rates compared to males, highlighting the gender-specific impact of these injuries.

Multidisciplinary collaboration is crucial for the comprehensive management of these patients. A holistic approach involving neurologists, psychiatrists, plastic surgeons, burn specialists, physical therapists, and mental health professionals is essential for addressing both the physical and psychological aspects of burn injuries in this population. Tailored medical interventions, ranging from initial wound care to advanced reconstructive procedures, must be coupled with long-term rehabilitation and psychological support to ensure the best possible outcomes for these vulnerable patients.

## References

[1] Miyoshi K, Morimura Y (2010). Clinical manifestations of neuropsychiatric disorders. Neuropsychiatric disorders.

[2] World Health Organization. (2021 (2021). Mental Disorders. https://www.who.int/news-room/fact-sheets/detail/mental-disorders.

[3] Shinoda J, Yokoyama K, Miwa K, Ito T, Asano Y, Yonezawa S, Yano H (2013). Epilepsy surgery of dysembryoplastic neuroepithelial tumors using advanced multitechnologies with combined neuroimaging and electrophysiological examinations. Epilepsy Behav Case Rep.

[4] Espinosa-Jovel C, Toledano R, Aledo-Serrano Á, García-Morales I, Gil-Nagel A (2018). Epidemiological profile of epilepsy in low income populations. Seizure.

[5] Boschini LP, Tyson AF, Samuel JC (2014). The role of seizure disorders in burn injury and outcome in Sub-Saharan Africa. J Burn Care Res.

[6] Newton CR, Garcia HH (2012). Epilepsy in poor regions of the world. Lancet.

[7] Unglaub F, Woodruff S, Demir E, Pallua N (2005). Patients with epilepsy: a high-risk population prone to severe burns as a consequence of seizures while showering. J Burn Care Rehabil.

[8] Spitz MC (1992). Severe burns as a consequence of seizures in patients with epilepsy. Epilepsia.

[9] Karacaoĝlan N, Uysal A (1995). Deep burns following epileptic seizures. Burns.

[10] Ibrahim A, Asuku ME (2014). Burns of the face in epilepsy: risk factors and an opportunity for prevention. Afr J Trauma.

[11] Mohammadi AA, Keshavarzi A, Erfani A, Modarresi MS, Shahriarirad R, Ranjbar K (2020). Evaluation of epilepsy and burn patterns in a tertiary hospital in southwestern Iran. Epilepsy Behav.

[12] Jang YC, Lee JW, Han KW, Han TH (2006). Burns in epilepsy: seven years of experience from the Hallym Burn Center in Korea. J Burn Care Res.

[13] Wang Y, Luo L, Li H (2024). Clinical profile, management and risk factors for seizure-related burn injuries among patients with epilepsy in southwest China. Heliyon.

[14] Zia Ziabari SM, Mobayen MR, Rimaz S, Nejat DR, Rimaz S (2022). Evaluation of patterns, cause and risk factors of burns in patients with seizure. Int J Burns Trauma.

[15] Li H, Yao Z, Tan J, Zhou J, Li Y, Wu J, Luo G (2017). Epidemiology and outcome analysis of 6325 burn patients: a five-year retrospective study in a major burn center in Southwest China. Sci Rep.

[16] Mackay FJ, Wilton LV, Pearce GL, Freemantle SN, Mann RD (1997). Safety of long-term lamotrigine in epilepsy. Epilepsia.

[17] Casillas-Espinosa PM, Powell KL, O'Brien TJ (2012). Regulators of synaptic transmission: roles in the pathogenesis and treatment of epilepsy. Epilepsia.

